# Attachment Style and Its Relationships with Early Memories of Separation Anxiety and Adult Separation Anxiety Symptoms among Emerging Adults

**DOI:** 10.3390/ijerph19148666

**Published:** 2022-07-16

**Authors:** Giulia Bassi, Elisa Mancinelli, Silvia Spaggiari, Adriana Lis, Silvia Salcuni, Daniela Di Riso

**Affiliations:** 1 Department of Developmental Psychology and Socialization, University of Padova, 35121 Padova, Italy; giulia.bassi@unipd.it (G.B.); silvia.spaggiari@studenti.unipd.it (S.S.); adriana.lis@unipd.it (A.L.); silvia.salcuni@unipd.it (S.S.); daniela.diriso@unipd.it (D.D.R.); 2 Digital Health Lab, Centre for Digital Health and Wellbeing, Fondazione Bruno Kessler, 38123 Trento, Italy

**Keywords:** emerging adulthood, adult romantic attachment, adult separation anxiety, retrospective memories of separation anxiety

## Abstract

Emerging adulthood concerns the transition from adolescence to adulthood. It foresees the separation from the family and the creation of new significant relationships, whereby specific attachment styles might be triggered when facing these challenges. The present study investigates the influence of retrospective memories associated with childhood separation anxiety symptoms upon emerging adults’ romantic avoidant vs. anxious attachment styles including adult separation anxiety symptoms as mediators. Age and gender were included as covariates. A community sample of N = 394 Italian emerging adults (Mage = 23.64, SD = 4.00, 70% females) completed self-report measures. The results showed that the participants presented a greater anxious attachment rather than an avoidant attachment style. Moreover, both adult separation anxiety and the memories of early separation anxiety were positively and significantly associated with anxious attachment and not with avoidant attachment. A mediation model conducted and focused on anxious attachment showed that, although not directly associated, child separation anxiety did show a significant positive indirect effect on anxious attachment as mediated by adult separation anxiety symptoms. Thus, the findings highlighted the influence of adult separation anxiety symptoms and retrospective childhood memories of separation anxiety upon anxious adult romantic attachment, yet not on avoidant attachment style. Clinical implications are discussed and suggestions for future research are provided.

## 1. Introduction

### 1.1. Romantic Attachment and Adult Separation Anxiety

Attachment theory has long acknowledged that the need to form and maintain close bonds is fundamental to humans throughout the course of life [1]. Attachment theory postulates that the early experiences of dyadic interactions with parents give rise to patterns of relational expectations, emotions, and behaviors regarding oneself and others that are embedded within the attachment system, then by guiding subsequent social competencies [1]. Bowlby [2,3] defined these cognitive schemas as Internal Working Models (IWM), which are mental representations resulting from two components: (1) a model of significant others (e.g., parents, close friends, and/or romantic partners), characterized by the expectations and beliefs concerning the availability, dependability, responsiveness, supportiveness, proximity, and comfort of attachment figures; and (2) a model of oneself, which includes information about whether one is worthy of attention, care, and support; one’s ability to get sufficient proximity/comfort; and one’s worth as a relationship partner. Once developed, these IWM operate as a guide to the development of later interpersonal relationships and specifically to how people think, feel, and relate to their close partners, as well as to the interpersonal world around them [4]. Accordingly, a distinction between secure, anxious, and avoidant attachment was made and then reflected in adult romantic attachment. Avoidant romantic attachment describes the degree to which individuals are uncomfortable with closeness and emotional intimacy in relationships. Specifically, avoidant individuals strive to create and maintain independence, control, and autonomy in their relationships [5], as they hold the belief that seeking psychological/emotional proximity with romantic partners is neither possible nor desirable. These beliefs motivate avoidant people to employ distancing strategies [6], whereby they defensively suppress negative thoughts and emotions to promote independence and autonomy. On the contrary, anxious romantic attachment concerns the degree to which individuals worry about being underappreciated or abandoned by their romantic partners. People experiencing heightened anxious attachment are heavily invested in their relationships, and they yearn for closeness with their partners in order to feel more secure [7]. Moreover, these individuals harbor negative self-views, while being both guarded and optimistic toward their romantic partners. These conflicting perceptions lead anxious individuals to question their worth, they worry about losing their partners, and they remain vigilant to signs that their partners might leave them [8,9]. Accordingly, people with anxious attachment are more prone to use emotion-focused and hyper-activating coping strategies when distressed, which sustain or escalate their worries and often keep their attachment systems chronically activated [10]. Avoidant and anxious attachments could thus play a role in facilitating or impeding adult tasks, which include dealing with separation from family and other attachment primary figures and facing attachment romantic bonds: during the challenging period of emerging adulthood, specific attachment patterns might be particularly critical as they are pivotal for social relationships [11].

### 1.2. Emerging Adulthood and Separation Anxiety

“Emerging Adulthood” is defined as a developmental period ranging approximately from 18 to 30 years of age and refers primarily to young adults who do not have children, do not live in their own home, or do not have sufficient income to become fully independent [12]. Although young adults are expected to leave home, which leads to further redefinitions of the relationship with their original family, about 50% of Italian young adults live at home up until the age of 35 and 30% are involved in long-distance romantic relationships, thereby delaying a stable commitment to a partner [13]. Indeed, the mean age of first marriages in Italy increased to 37.91 years of age in 2017 [14]. Moreover, in the Italian context, the family is the main care and welfare provider for emerging adults, who are facing a transition towards the uncertainty of a labor market, that is exacerbated by crucial social policies. Thus, they are most often financially sustained by their parents, that means permitting them to more easily attend school and/or find a job, resulting in prolonged co-residence [15]. Moreover, culturally bound psychosocial factors play a role in delaying young adults’ separation from their parents. The literature reports that both parents and their emerging adult offspring perceive living together as a positive experience [16,17]. So, young people, still living at home with their parents, then demonstrated difficulty in becoming independent and in taking on responsibilities [18], because they relied on their comfort zone. Although chronologically adulthood has been achieved, adult psychosocial maturity has not, thus this period can be considered as a delay in reaching developmental tasks, leading to difficulty in facing adult responsibilities and romantic relationships [19]. Accordingly, the prolongation of this transition can become “a combined developmental undertaking” [20] for both parents and their children. Therefore, emerging adulthood can become a phase of uncertainty, so much so that the term “feeling in-between” was adopted to describe this [17]. Indeed, emerging adults may struggle with identity issues, with difficulty in finding a place in the adult world, and in making satisfying choices regarding their romantic relationships and work environment, all of which can subsequently result in experiencing symptoms of anxiety [21].

In this regard, it is noteworthy that the fifth edition of the *Diagnostic and Statistical Manual of Mental Disorders* (DSM-5) [22] has included the Adult Separation Anxiety Disorder (ASAD), thereby acknowledging that adulthood can be a potential period in which separation anxiety may appear and thus it might play a role across one’s whole lifespan. ASAD is characterized by an intense fear of actual or potential separation from close attachment figures and a consequent worry regarding the safety and whereabouts of such persons [22]. Among young adults, anxiety symptoms might be experienced when they separate from their parents or intimate partners, or when they are faced with life changes, such as moving or getting married [22]. The literature shows that separation anxiety correlates with a reduced differentiation of oneself, and it hardly impacts different aspects of life, such as work, close relationships, and social and private leisure activities [23]. However, genetic, familial, bonding, and attachment factors may be involved in the genesis of ASAD [24]. The changes introduced by the DSM-5 have led to increased research into the study of ASAD regarding its epidemiology, prevalence, age of onset [25,26], correlates [27], and comorbidities [25,28], with a mostly categorical approach. As emerged from epidemiological studies, childhood Separation Anxiety Disorder (SAD) may persist into adulthood, suggesting continuities between SAD and ASAD [29]. However, other studies also support the onset of separation anxiety symptoms in adulthood [26]. In attachment theory, the construct of separation anxiety has always played a pivotal role. From an evolutionary point of view, the attachment system, beyond other roles, promotes survival by maintaining proximity between parents (or other caregiving figures) and children, which allows them to experience reduced symptoms of fear, anxiety, and distress-related symptoms when experiencing separation. Accordingly, heightened anxiety symptoms associated with separation from the primary caregiver can be an indicator of insecure attachment, thereby referring to issues related to the child’s IWM as shaped by previous and ongoing bonding experiences with the caregiver [2,3]. Research on adult separation anxiety symptoms and, for instance, current studies relevant to the COVID-19 psychological impact, suggest that relying on adults’ memories can be considered valid and reliable, in particular when investigating subjective experiences rather than specific behavioral manifestations or detailed instances of separation anxiety [30,31]. Studies on early memories of separation anxiety symptoms are mainly focused on the connection with the developmental trajectories of SAD, while the investigation of the link between early memories of separation anxiety symptoms and adult romantic attachment styles is still in its infancy.

Notably, all of the aforementioned literature regarding separation anxiety focuses on the identification of early memories of separation anxiety symptoms and/or adult separation anxiety in clinical samples. Very little attention has been given to community samples whereby an ASAD clinical diagnosis is not considered a milestone [26,32]. To our current knowledge, no studies have tried to analyze the peculiarity of emerging adulthood, by distinguishing between retrospective memories of separation anxiety symptoms and adult separation anxiety symptoms according to a dimensional perspective, while also investigating their association with romantic attachment. Few papers have focused on these issues, and those that did have concentrated on research mainly carried out in clinical populations [28,33].

### 1.3. Objective

The present study is aimed at exploratively investigating how retrospective memories of separation anxiety symptoms and adult separation anxiety can be associated with emerging adults’ avoidant and anxious attachments. In this regard, the first aim is to investigate which romantic attachment style (anxious vs. avoidant) is prevalent among Italian emerging adults. The second aim is to evaluate how romantic attachment is associated with adult separation anxiety profiles based on the symptom levels observed in adult separation anxiety as well as the retrospective memories of separation anxiety symptoms. Last, the influence of retrospective memories associated with early memories of separation anxiety symptoms upon emerging adults’ avoidant vs. anxious romantic attachment styles, including adult separation anxiety symptoms as mediators, is explored.

## 2. Methods

### 2.1. Procedure and Participants

A total of N = 394 Italian emerging adults participated in the study, of which 276 (70%) were female and aged between 18 and 30 years (Mage = 23.64, SD = 4.00). The participants were all university students (undergraduate and postgraduate). All participants indicated that they had not been hospitalized with symptoms of psychiatric disorders in the past two years. A strong minority (<3% of the total sample) reported previous psychological counselling or intervention in the past two years for mild problems, unrelated to separation anxiety problems. Only 34 participants (4.1%) were in a common-law relationship and only one (3.6%) was married. The study procedure was conducted in compliance with the Declaration of Helsinki (Italian law 196/2003, UE GDPR 679/2016) and approved by the Ethical Interdepartmental Committee of Padova University (1266/2013). The participants provided their consent prior to their participation. All were informed that their data were confidential, that they could refuse to disclose information they did not want to provide, and that they could withdraw from the study at any moment. Trained master’s students assisted the participants in the compilation of the self-report measures during regular university hours.

### 2.2. Measures

#### 2.2.1. Separation Anxiety Symptom Inventory

The Separation Anxiety Symptom Inventory (SASI) [29] is a 15-item self-report questionnaire, that captures the retrospective memories of separation anxiety symptoms and their frequency prior to/up until the age of 18. It generates dimensional rather than categorical responses. Items are rated on a four-point Likert scale (from 0 = “this has never happened” to 3 = “this happens very often”). Individual items are summed up to obtain a total score for SASI, which are considered in the current study. Higher scores indicate greater retrospective memories of separation anxiety symptoms in childhood. The SASI has been translated and validated in Italy [34]. In the present study, the internal consistency shows α = 0.83 (95% CI = [0.80, 0.85]).

#### 2.2.2. Adult Separation Anxiety–27

The Adult Separation Anxiety–27 (ASA-27) [35] is a 27-item self-report inventory, which is designed to assess separation anxiety symptoms in adulthood. Items are rated on a 4-point Likert scale (from 0 “this has never happened” to 3 “this happens very often”). Scores across items are summed up to obtain a total score for ASA-27, which are considered in the current study. Higher scores indicate a greater severity of adult separation anxiety symptoms. The ASA-27 has been translated and validated in Italy on a sample of Italian university students, showing good psychometric properties [36]. In the present study, the ASA-27 shows a satisfactory internal consistency of α = 0.89 (95% CI = [0.89, 0.91]).

#### 2.2.3. The Experiences in Close Relationships Questionnaire-Revised

The Experiences in Close Relationships Questionnaire-Revised (ECR-R) [37] is a 36-item measure of adult romantic attachment style. The statements concern how the individual feels in emotionally intimate relationships, and how they generally experience relationships, not merely what is happening in a current relationship. More specifically, the ECR-R is designed to assess individual differences regarding two dimensions of attachment (18 items for each scale). The first dimension refers to attachment-related anxiety (ECR-Anxiety), which indicates the extent to which people are insecure vs. secure about the availability and responsiveness of romantic partners. The second dimension refers to attachment-related avoidance (ECR-Avoidance), which designates the extent to which people are uncomfortable being close to others vs. secure depending on others. Both dimensions are considered in the current study. Each item is rated on a 7-point Likert scale (from 1 “strongly disagree” to 7 “strongly agree”). Higher mean scores indicate greater degrees of anxious and/or avoidant romantic attachment. The ECR-R has been translated and validated in Italy [38]. In the present study, the internal consistency is α = 0.90 (95% CI = [0.89, 0.91]) for ECR-Anxiety, and α = 0.92 (95% CI = [0.91, 0.93]) for ECR-Avoidance.

### 2.3. Data Analysis

The analyses were performed using R and SPSS v.20. The preliminary analysis regarded demographic (N; %) and descriptive information (Median, Quartile) and the assessment of the variables’ normal distribution through the Shapiro-Wilk test. The Shapiro-Wilk test was used to assess the distribution of the present variables (i.e., SASI, ASA-27, ECR-Anxiety, ECR-Avoidance). All tests were significant (*p* < 0.00), indicating that none of the variables presented a normal distribution.

Subsequently, the sample level of anxious (ECR-Anxiety) vs. avoidant (ECR-Avoidance) attachment was compared using the Wilcoxon test for paired samples. Furthermore, to explore the variables’ associative patterns, Spearman rho correlations were performed and interpreted when Cohen’s d effect size was at least medium (>0.30) [39]. All analyses were considered significant when *p* < 0.05.

A mediational model (PROCESS Model 4) [40] was performed including SASI as the criterion variable and ECR-Anxiety as the dependent variable with ASA-27 as the mediator. Age and gender were included as covariates. The bootstrapping method of drawing 5000 bootstraps was then applied. The effects’ significance was assessed based on a 95% Confidence Interval (CI); the effects were considered significant when the CI excluded 0. It should be noted that the PROCESS’ mediational models adopt the Ordinary Least Square (OLS) regression method to estimate the models’ effects [40], meaning that associations between variables are assessed while controlling for the influence of all others included in the model. Through the OLS regression method, it is thus possible to control for the variables’ shared variance, thereby justifying performing mediational analysis on cross-sectional data in which no causal relations can be implied. Accordingly, terms such as “effect”, “influence”, or “mediate” will be used in line with the model performed, but not to suggest causality.

## 3. Results

### 3.1. Preliminary Analysis

The descriptive information is reported in Table 1. The participants consisted of N = 394 Italian emerging adults aged between 18 and 30 years (Mage = 23.64, SD = 4.00, 70.1% females). At the time of the study, most of the participants were university students (n = 385, 97.72%), and only n = 9 (2.3%) were university students in paid employment. With regard to the sample relationship status, the great majority of the participants (n = 347, 88.1%) were single, n = 23 (5.8%) were cohabitating with their partner, and only n = 14 (3.6%) were married. Ten (n = 10, 2.5% did not provide any information).

The Wilcoxon test for paired samples was performed to investigate differences in the participants’ levels of ECR-Anxiety vs. ECR-Avoidance. The results showed that the current sample presented significantly higher scores for ECR-Anxiety compared to those for ECR-Avoidance (stat = 9630; *p* < 0.00).

### 3.2. Correlations

The correlations are shown in Table 2, highlighting a significant negative association between the participants’ age and all of the considered variables, although all show a small effect size. ECR-Anxiety presented positive and significant correlations with ASA-27 and SASI with medium effect sizes, while ECR-Avoidance showed negligible or insignificant correlations with ASA-27 and SASI. ECR-Anxiety and ECR-Avoidance were positively and significantly correlated with medium effect sizes, while SASI and ASA-27 were positively and significantly correlated with a large effect size.

**Figure 1:**
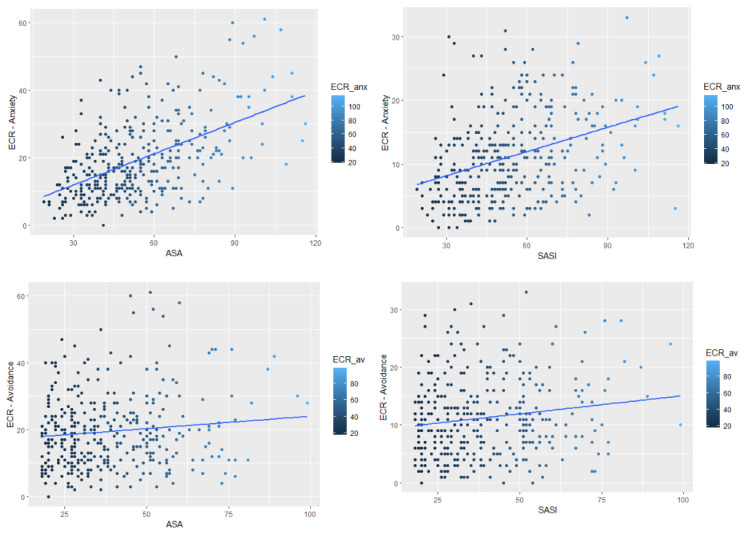
Scatterplot of the linear association between both ECR-Anxiety and ECR-Avoidance and ASA-27 and SASI.

To further explore and to visually inspect the correlational results, four plots displaying the distribution and the linear associations between ECR-Anxiety and both ASA-27 and SASI (Figure 1, top plots), as well as the associations between ECR-Avoidance, ASA-27, and SASI (Figure 1, bottom plots) have been produced.

### 3.3. The Mediational Model

**Figure 2:**
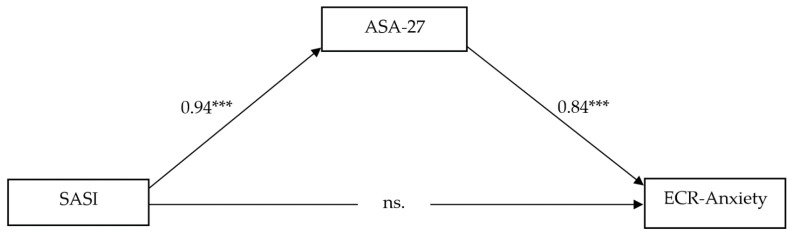
Mediational model. *** *p* < 0.00. ns = non-significant.

In line with the obtained correlational results, only one mediational model was performed in order to investigate the direct and indirect effects of SASI on ECR-Anxiety and mediated by ASA-27, as displayed in Figure 2. Since neither SASI nor ASA-27 has been shown to significantly contribute to ECR-Avoidance, no mediational model with these variables was performed. Moreover, as reported in the Data Analysis section, only effect size medium (>0.30) results were considered in the present study.

The overall model comprising all criterion variables (i.e., SASI and ASA-27) as well as the covariates (i.e., age and gender) accounted for the 13.67% of ECR-Anxiety total variance. This model highlighted a significant total effect (β = 0.95; t = 6.96; CI = 0.68, 1.22).

In particular, as shown in Figure 2, SASI showed a significant positive association with ASA-27 (β = 0.94; t = 13.65; CI = 0.81, 1.08) but no significant direct effect on ECR-Anxiety. On the other hand, ASA-27 showed a significant positive association with ECR-Anxiety (β = 0.84; t = 9.23; CI = 0.66, 1.02). Accordingly, although not directly associated, ASA-27 showed a mediating role in the significant indirect association between SASI and ECR-Anxiety (β = 0.79; CI = 0.56, 1.04). Age and gender, when included as covariates, were significant when assessing SASI and ASA-27 effects on ECR-Anxiety, suggesting that both age and gender significantly contribute to these associations. On the other hand, only gender emerged as a significant covariate when calculating the association between SASI and ASA-27, suggesting significant gender differences in this association.

## 4. Discussion

The present study aimed at exploratively investigating how retrospective memories of separation anxiety symptoms and adult separation anxiety could be associated with emerging adults’ romantic avoidant versus anxious attachment styles. Few studies have investigated these relationships and those that did, were mainly focused on clinical populations [28,33]. Thus, little attention has been paid to the associations of the above-mentioned variables in a community sample, in particular among Italian emerging adults. Following Silove and colleagues’ studies [28], the present study adopted a dimensional perspective instead of a categorical approach in the interpretation of the results that emerged from the analyses.

The results related to the first and second aims of the present study pointed out that emerging adults showed a significantly higher anxious romantic attachment rather than an avoidant romantic attachment style. However, a positive significant correlation was found between anxious and avoidant attachment styles, which can indicate an increase in the use of anxious strategies as well as an increase in the use of avoidant strategies in romantic attachment styles. The results showed that early memories of separation anxiety and separation anxiety symptoms in adulthood are related to a more anxious attachment style. These findings are further supported by the plots showing the linear association between variables, which highlight how emerging adults with anxious romantic attachment are distributed evenly regarding both separation anxiety symptoms and early memories of separation anxiety. This distribution was not observed for avoidant attachment, which has no significant association with separation anxiety symptoms among emerging adults. Therefore, no mediation model was performed considering avoidance attachment as the dependent variable, whereas it was carried out considering anxious attachment and including both adults’ reported separation anxiety symptoms and early memories of separation anxiety. This latter mediational model, as the third aim of the present study, shows that early memories of separation anxiety symptoms influence anxious romantic attachment, only as a contribution to the presence of separation anxiety symptoms. In other words, the more that young adults report early memories of separation anxiety and report having experienced adult separation anxiety symptoms, the more they display an anxious attachment style in their close relationships. These results are consistent with the available literature regarding the developmental challenges that emerging adults have to face, characterized, in particular, by the experience of “feeling in-between” [17]. Emerging adults have to face a process of exploration in several key areas of their lives, such as the search for a romantic relationship, which represents one of their intrinsic developmental tasks [9]. However, in the Italian context, this exploration usually occurs within the parents’ household, where most emerging adults continue to live [13], thereby relying on their family nucleus as their safe zone even in adulthood. The process of leaving the parents’ house represents a real-life separation experience for young adults, thereby delaying taking responsibility and their commitment to romantic relationships [19]. The experience of increased adult separation symptoms can increase difficulties in developing romantic attachment relationships and, as a consequence, increase the chances of adopting an anxious attachment style [21]. Indeed, it is in this context that the way an individual responds behaviorally and emotionally to close relationships is influenced by their mental representations of attachment relationships, developed through experiences with significant others [9] (i.e., early memories of separation anxiety). In line with this, Brennan’s model suggests that adults with high levels of romantic anxious attachment overvalue their relationships, show a negative image of themselves, and fear separation [9]. Therefore, this mediational model may provide additional knowledge to the literature, which, in turn, can be useful for clinical practice to understand the importance of tracing back retrospective memories of separation anxiety experienced during childhood to influence current adult separation anxiety symptoms. Moreover, this pattern can guide clinicians and researchers in gaining an in-depth understanding of the way emerging adults relate to romantic partners, particularly when an anxious attachment style is involved. To conclude, these results suggest the presence of a possible continuation between early memories of separation anxiety symptoms and adult separation anxiety symptoms in emerging adults with anxious romantic attachment, in which the separation–individuation process is one of the main challenges regarding this transitional phase. With regard to the limitations, the present study used a cross-sectional design and thus the presence of a cause–effect relationship between the considered variables cannot be inferred. Furthermore, only retrospective memories of separation anxiety symptoms among emerging adults were considered; indeed, the literature should focus on the development of longitudinal studies in order to investigate the trajectory of the associations between these variables from childhood to adulthood. Moreover, the self-report questionnaires administered may not reveal the unconscious features of emerging adults’ romantic attachment, and thus, future studies should combine quantitative data with the use of semi-structured interviews, such as the Adult Attachment Interview [41,42], which has already been used as a tool to predict behavior in romantic relationships [43,44]. Notwithstanding these limitations, the mediational model that emerged from this study may help in the understanding of the underlying challenges that this life period brings, specifically with regard to the influence of adult separation anxiety symptoms in the relationship between early memories of separation anxiety and anxious romantic attachment.

## 5. Conclusions

Extensive research has explored the impact of early experiences, particularly within the family [45,46], upon adult development, including personal adjustment [47] and attachment ties [48]. The instruments administered to adults focus on their current development, while early experiences are investigated, posing the same questions to both children and adolescents, yet with the items phrased using the past tense [49,50]. Therefore, early influences on adults’ current adjustment can be studied and investigated consistently without the need for costly longitudinal studies [49,50] but instead relying on the use of instruments such as SASI. Following this line, to our knowledge, the current study is the first to investigate how childhood memories of separation anxiety symptoms and adult separation anxiety may be linked to the romantic avoidant versus anxious attachment styles among emerging adults. This study highlights how early memories of separation anxiety symptoms influence how emerging adults interact in romantic relationships, only when they report experiencing separation anxiety symptoms in adulthood. In this regard, one can speculate about a possible continuation between early memories of separation anxiety symptoms and adult separation anxiety symptoms in emerging adults, in particular within the context of anxious romantic attachment. Therefore, this research adds knowledge on the importance of supporting young adults in the process of separation–individuation and on the importance of establishing healthy romantic relationships, which is one of the main developmental tasks of emerging adulthood. In this transitional period, the role of the family, both directly and indirectly, continues to be a key developmental component for young adults, which should be considered in the implementation of future works.

## Figures and Tables

**Table 1:** Variables’ median and values range within each quartile.

	*Q*1 *Md* (*R*)	*IQR* *Md* (*R*)	*Q*3 *Md* (*R*)
**ECR-Anxiety**	35 (19–40)	52 (41–66)	78 (67–116)
**ECR-Avoidance**	22 (18–27)	36 (28–50)	59 (51–99)
**SASI**	4 (0–6)	10 (7–15)	20 (16–33)
**ASA**	8 (0–11)	17 (12–24)	31 (25–61)

**Note.***Md* = median; *R* = Range; *IQR* = *Q*3–*Q*1.

**Table 2:** Spearman’s Rho correlations.

	Age	ECR-Anxiety	ECR-Avoidance	ASA	SASI
**ECRanx**	−22 *	—			
**ECRav**	−12 *	0.45 **	—		
**ASA**	−24 *	0.49 **	0.08	—	
**SASI**	−24 *	0.38 **	0.13 *	0.61 **	—

**Note.** * *p* < 0.05; ** *p* < 0.01.

## Data Availability

The data presented in this study are available on request from the corresponding author. The data are not publicly available due to data privacy.
